# Supplementary material for: Learning curve and surgical outcome of robotic assisted colorectal surgery with ERAS program
Source: Sci Rep. 2022 Nov 29;12:20566. doi: 10.1038/s41598-022-24665-w (PMC9709162; doi:10.1038/s41598-022-24665-w)
Supplement: Supplementary file 1 — Supplementary Information 1. [file 41598_2022_24665_MOESM1_ESM.zip › LSC Raw data-Ñ[▒K/065/P_ñw╝╨░O▒Kñσ.pdf]

[redacted]

Pathologic diagnosis:
1. Sigmoid colon, laparoscopic anterior resection --- a. Status post endoscopic mucosal resection at outside hospital. No tumor seen. The bilateral cut ends and mesocolic lymph nodes (0/12) are unremarkable.
b. Hyperplastic polyp.
2. IMA root, dissection --- Unremarkable lymph node (0/1).
3. Colon, cut end, distal, resection --- Unremarkable.

Gross description:
The specimen consists of 1) a segment of colon, 23.6 cm in length and 6.5 cm in proximal cut end circumference, 5.1 cm in distal circumference, and mesocolic soft tissue. A surgical stitch labeled post-EMR scar, 0.5 cm, is noted, 4 cm to the distal cut end. There is no tumor found. The both cut ends are free of tumor grossly. 2) IMA root, a piece of tan yellow soft tissue, 3x1.2x0.9 cm. 3) distal cut end, a piece of tan mucosa-covered soft tissue, 2.5x2.5x1 cm.
Representative part for section: A) proximal cut end B) distal cut end C-D) EMR scar E) radial margin (inked yellow) F) all of specimen 2 G) specimen 3 H-P) lymph nodes.
#T-59300_2 #M-09413_2 0 1105 SP0000
#T-C4560_2 #M-09183_2 0 1105 000000

已電子簽章

敬告：本單僅供醫師參考使用，不具任何申請效力！！
請病患妥為保管，若不慎遺失需自負個人資料外洩責任！
